# Time restricted feeding is associated with poor performance in specific cognitive domains of Suburb-Dwelling older Chinese

**DOI:** 10.1038/s41598-022-23931-1

**Published:** 2023-01-08

**Authors:** Jiayu Li, Ruijin Li, Xuan Lian, Peipei Han, Yuewen Liu, Chuanchuan Liu, Beibei Wang, Chunliu Xu, Feng Wang, Jingru Wang, Xueling Zheng, Mingyao Shen, Yanan Zha, Bin Mao, Ming Li, Ying Yu, Qi Guo

**Affiliations:** 1 Department of Rehabilitation Medicine, College of Rehabilitation Sciences, Shanghai University of Medicine and Health Sciences Affiliated Zhoupu Hospital, 279 Zhouzhu Highway, Pudong New Area, Shanghai, 201318 China; 2 Shanghai University of Traditional Chinese Medicine, Shanghai, China; 3 University of Shanghai for Science and Technology, Shanghai, China; 4 Baozhen Community Health Service Center, Chongming District, Shanghai, China; 5 Department of Rehabilitation Medicine, Shanghai University of Medicine and Health Sciences, Shanghai, China

**Keywords:** Medical research, Risk factors

## Abstract

The purpose of this study was to investigate the association between time restricted feeding (TRF) and different areas of cognitive function in the elderly in Chinese communities. This study consisted of 1353 community-dwelling Chinese older adults aged 60 years and older in Chongming area, Shanghai (563 males; the mean age, 73.38 ± 6.16 years). Mild cognitive impairment (MCI) and six different cognitive domains was assessed by the Chinese-version of Mini Mental State Examination (MMSE). Recording the eating time of each meal through oral inquiry to calculate the time window between the first meal and the last meal of the average day. Participants with an eating time window duration of more than 10 h were then identified, as well as those with eating time restricted to less than 10 h (TRF). Our study found that TRF may be associated with a higher incidence rate of cognitive impairment. TRF only limited the eating time window and did not change the frequency of participants' dietary intake. We used a linear regression model to study the association of TRF with cognitive function. After adjusting for confounding variables, the results showed that TRF was related to MMSE score (*P* < 0.001), "Orientation to place" (*P* < 0.001) and "Attention/calculation" (*P* < 0.001) functions. Among Chinese older community-dwellers, TRF was associated with a higher prevalence of CI and negatively correlated with the "Orientation to place" and "attention/calculation" functions.

## Introduction

Mild cognitive impairment (MCI) is a state of cognitive function between that seen in normal aging and dementia, resulting in cognitive problems in many fields, including difficulties in memory, language, attention, orientation, calculation, visuospatial ability and executive functions^1,2^. In China, the prevalence of MCI is 40.0% in males and 45.1% in females^3^. Recent evidence showed that dietary behaviors might have a direct effect on brain function, in which the correct eating time played an important role in healthy life span and might further affect cognitive status^4,5^.

As one of intermittent fasting (IF), time restricted feeding (TRF) consolidates all calorie intake to 6–10 h periods during the active phase of the day, without necessarily changing diet quality and quantity^6^. At present, most studies of TRF and cognitive status have been conducted in the laboratory environment, the relationship between TRF and MCI is inconclusive. In a cross-sectional study involving 883 older Italian adults, Currenti and colleagues found that individuals adherent to 10-h TRF were less likely to have CI, compared to those with no eating time restrictions^7^. TRF may play a role in the regulation of behavior and modulation of the expression of brain derived neurotrophic factor (BDNF), so as to influence neurogenesis and cognitive function^8^. However, an 8-week pilot clinical trial showed that IF did not change cognitive performance^9^. TRF may limit calorie intake throughout the day, increasing the risk of weakness and cognitive impairment in the general elderly^10–12^. Due to the different study populations and methods, there were contradictions in the existing research results; In addition, these studies did not involve the impact of TRF on specific cognitive domains. The results of laboratory mouse models suggested that IF might have different effects on different cognitive fields, but there was still a lack of relevant human research^13,14^. In addition, due to the complexity of the specific mechanism of TRF, many factors, including circadian rhythm and age, may also cause the difference between the results^15,16^. These are worthy of in-depth discussion in the research.

Although TRF has shown improvement in cognitive function in the most of previous 6–8 h studies, these studies reported several adverse events, including vomiting, headache, increased thirst and diarrhea, which were not found in the 10 h TRF study^7–10^. In view of the above, we hypothesized that compared with the 6–8 h TRF under normal circumstances, controlling the "time window" of daily eating within 10 h might be more applicable in the elderly population. Therefore, we conducted a cross-sectional study to analyze the association of 10-h TRF with different cognitive domains of the elderly in Chinese communities, and to explore the potential influencing factors leading to these differences.

## Methods

### Study participants

Our research population included residents from Chongming District, Shanghai, who had joined China’s national free physical examination program from August 2019 to July 2020. The physical examination data of the elderly living in four different communities were collected, including demographic and health-related parameters. Inclusion criteria were: (1) 60 years of age or older at baseline; (2) Have lived in the community for at last one year; (3) Willing to participate baseline studies. The exclusion criteria for this study were: (1) Severe cognitive impairment, dementia, mental illness or other neurodegenerative diseases; (2) Those who could not take care of themselves and could not walk independently; (3) People who unable to complete the questionnaire due to hearing impairment, visual impairment and communication difficulties. Following these criteria, the final study populations comprised 1353 subjects.

All participants were fully informed of the nature of research and signed an informed consent prior to participate in. This study was conducted in accordance with national and international norms and the recommendations of our ethics committee, and was approved by the Ethical Committee of the Shanghai University of Medicine and Health Sciences.

### Dietary assessment

Our study used Food Frequency Questionnaire (FFQ) to investigate food intake, which was formulated by the National Institute for Nutrition and Health Chinese Center for Disease Control and Prevention. On this basis, our study adapted it combined with the research purpose. FFQ can investigate the food intake of the subjects in the past period of time. We assessed the frequency of main dietary intake of participants over the past month and divided them into 13 food groups.

### Time feeding

This study mainly investigated the eating time of participants in the month before the survey. Participants were asked whether and when they ate daily meals (including breakfast, lunch and dinner), in order to calculate the time window between the first meal and the last meal of the average day. The 10-h eating window determined based on the start time of the first meal and the end time of the last meal. And finally, participants were divided into those eating time window lasted more than 10 h and those whose eating time was limited to less than 10 h (TRF).

### Cognitive status

We assessed the cognitive function of the participants on the survey day (i.e., one month after the participants adhered to their eating time). Cognitive function is assessed by the Chinese-version of the Mini Mental State Examination (MMSE), a simple cognitive impairment screening test that can be used to assess multiple cognitive domains. The highest total score is 30, with the higher score indicates the better status. We divided these items into six areas to measure different cognitive processes: orientation to time (5 points), orientation to place (5 points), registration (3 points), attention and calculation (5 points), recall (3 points) and language (9 points)^17^. Considering the educational level of participants, the cut-off points of CI were as follow: ≤ 17 for illiterate, ≤ 20 for people graduated from primary school, and ≤ 24 for people with middle school education or above^18^.

### Covariates

As part of the National Free Medical Examination Program, after completing the free physical examination, all participates were invited to conduct face-to-face interviews to answer a standardized questionnaire. The questionnaire included questions about age, gender, height, body weight, illiterate, living alone, sleeping duration, widowed, farming, smoking habit (non-smoker, former smoker and current smoker) and alcohol consumption (never drinker, former drinker, occasional drinker and current drinker). Living scale (IADL) judged normal daily activity ability. IADL includes 8 items, with scores ranging from 0 to 8, which scores ≥ 6 indicates normal daily activity ability^19^. We used the short form of the International Physical Activity Questionnaire (IPAQ) to assess physical activity^20^. Data regarding personal history of physical illness assessed through past diagnoses made by physicians and current or historical medication regimens was also collected. Diseases of interest included type 2 diabetes mellitus, hypertension, hyperlipidemia, stroke, heart disease and cancer, etc.

### Statistical analysis

IBM SPSS v25.0 software (SPSS Inc., Chicago, IL, USA) was used for all statistical calculations. The significance level in the current study was set at 0.05. We analyzed the baseline cross-sectional data of this population. Categorical variables are expressed in occurrence and percentages; Chi-squared test was used for the difference between groups, *φ* indicated effect sizes. Continuous variables are expressed as means ± standard deviation; continuous nonnormal distribution variables, such as IPAQ, were expressed as median and quartile; differences between groups were tested by Student’s t-test or Mann–Whitney U-test for normal and non-normal distribution variables, Cohen’s d indicated effect sizes. A Poisson regression model was used to analyze the associations between potential correlates and the participation in TRF groups, which resulted in a prevalence ratio (PR) for each variable. All multivariable analyses were adjusted for socio–demographic and health–related covariates (Model 1: age, sex, BMI, IADL, illiterate, widowed, living alone, farming, smoking, drinking, night-time feeding, IPAQ, type-2 diabetes, hypertension, dyslipidemia, stroke, heart disease, and cancer). The Multiple linear regression model evaluated the association between time restricted feeding and cognitive domains (time, place, registration, recall, attention and calculation, and language), and adjusted according to the above contents.

### Ethical standards

This study was approved by the Shanghai University of Medicine and Health Sciences ethics committee.

## Results

### Baseline characteristics

Of the 1353 participants in the final analysis, 41.6% (n = 563) were men. The average age of the subjects was 73.19 ± 6.10 years old, more than half of them were engaged in manual labor, and the education level of most subjects was at or below primary school. Table 1 presents the background characteristics according to the duration of the eating time window (TRF is the control group). In contrast, among those having TRF were older (*P* = 0.017, *φ* = 0.21) and were more likely to suffer from hypertension (*P* = 0.012, Cohen’s d = 0.17) and stroke (*P* = 0.033, Cohen’s d = 0.21). In addition, participants with eating time window lasted more than 10 h had higher daily activity ability and level (*P* = 0.021, Cohen’s d = − 1.04) but were less likely to be farming (*P* = 0.030, Cohen’s d = 0.16). Among participants in TRF, the prevalence of CI was higher than that in the no restriction group (*P* < 0.001, *φ* = 0.33). Baseline data showed that the MMSE score (*P* < 0.001, Cohen’s d = − 0.57), as well as scores in orientation to place (*P* < 0.001, Cohen’s d = − 1.04) and attention and calculation (*P* < 0.001, Cohen’s d = − 0.79) were lower in the TRF group.

**Table1:** Background characteristics of the study population according to eating time window duration (time feeding restricted to 10 h vs. no restriction).

Variables	TRF* (n = 409)	No restriction (n = 944)	Cohen’s d /*φ*	*P*
**Age group (years)**			0.21	0.017
60–64	12 (2.9)	35 (3.7)		
65–74	228 (55.7)	595 (63.0)		
≥ 75	169 (41.3)	314 (33.3)		
**Sex (%)**			0.03	0.270
Male	161 (39.4)	402 (42.6)		
Female (%)	248 (60.6)	542 (57.4)		
BMI (kg/m^2^)	23.92 ± 3.46	23.61 ± 3.33	0.09	0.313
IADL (score)	6.57 ± 1.14	7.68 ± 1.00	− 1.04	0.021
MNA-SF (score)	12.89 ± 1.35	12.82 ± 1.45	0.05	0.163
GDS (score)	13.06 ± 3.34	12.70 ± 3.24	0.11	0.334
Sleep duration (hour)	8.48 ± 1.34	8.35 ± 1.33	0.10	0.471
IPAQ (Met/week)	4746 (2039.25,8484)	4893 (2373,9786)	− 0.12	0.107
Living alone (%)	77 (18.8)	161 (17.1)	0.02	0.432
Widowed (%)	93 (22.7)	184 (19.5)	0.04	0.174
Illiterate (%)	62 (15.2)	109 (11.5)	0.07	0.066
Farming (%)	214 (52.3)	554 (58.7)	0.16	0.030
**Smoking (%)**			0.06	0.129
Non-smoker	301 (73.6)	644 (68.2)		
Former smoker	56 (13.7)	163 (17.3)		
Current smoker	52 (12.7)	137 (14.5)		
**Drinking (%)**			0.06	0.204
Never drinker	262 (64.1)	588 (62.3)		
Former drinker	51 (12.5)	92 (9.7)		
Occasional drinker	38 (9.3)	113 (12.0)		
Current drinker	58 (14.2)	151 (16.0)		
**Health status (%)**				
Type-2 diabetes	67 (16.4)	167 (17.7)	0.02	0.559
Hypertension	279 (68.2)	576 (61.0)	0.17	0.012
Hyperlipidemia	58 (14.2)	120 (12.7)	0.02	0.463
Stroke	138 (33.7)	264 (28.0)	0.21	0.033
Heart disease	145 (35.5)	329 (34.9)	0.01	0.832
Cancer	12 (3.0)	34 (3.6)	0.02	0.569
**The time of food intake (%)**			1.13	< 0.001
Daytime feeding	388 (94.9)	808 (85.6)		
Night-time feeding	21 (5.1)	136 (14.4)		
CI (%)	144 (35.2)	137 (14.5)	0.33	< 0.001
MMSE (score)	22.45 ± 4.63	24.97 ± 4.24	− 0.57	< 0.001
Orientation to time (score)	4.21 ± 1.15	4.30 ± 1.00	− 0.08	0.540
Orientation to place (score)	3.65 ± 0.83	4.73 ± 0.71	− 1.40	< 0.001
Registration (score)	2.75 ± 0.52	2.85 ± 0.51	− 0.19	0.658
Attention/calculation (score)	2.36 ± 1.64	3.64 ± 1.60	− 0.79	< 0.001
Recall (score)	1.35 ± 1.27	1.34 ± 1.23	0.01	0.918
Language (score)	8.04 ± 1.27	8.10 ± 1.18	− 0.05	0.603

*TRF* Time restricted feeding, *BMI* Body Mass Index, *IADL* Instrumental activities of daily living, *MNA-SF* Mini-nutritional assessment short-form, *GDS* Geriatric Depression Scale, *IPAQ* International physical activity questionnaires, *CI* Cognitive impairment, *MMSE* Mini-mental State Examination.

*TRF is the control group. ^△^Cohen’s d indicates effects sizes in Student’s t-test and Mann–Whitney U-test, φ indicates effects sizes in Chi-squared test.

### Dietary intake frequency of the study population

In this study, most participants used to daytime feeding, while in the TRF group, the proportion was much higher (*P* < 0.001, *φ* = 1.13, Table 1). Table 2 shows the frequency of food intake across the TRF group and no restriction group. In terms of daily diet, except for the lower intake frequency of the TRF group in terms of main food (*P* < 0.001, *φ* = 0.11), there was no significant difference in dietary intake frequency between TRF group and no restriction group in other food groups (All *P* > 0.05).

**Table 2:** The frequency of major food intake according to feeding time window duration (time feeding restricted to 10 h vs. no restriction).

Variables	TRF* (n = 409)	No restriction (n = 944)	*φ*	*P*
**Main food (%)**			0.11	< 0.001
≥ 3 times/day	382 (93.4)	920 (97.5)		
≤ 2 times/day	27 (6.6)	24 (2.5)		
**Vegetable and fruit (%)**			0.01	0.778
≥ 2 times/day	384 (93.9)	890 (94.3)		
< 2 times/day	25 (6.1)	54 (5.7)		
**Coarse cereals (corn) (%)**			0.05	0.286
≥ 1 time/day	96 (23.5)	225 (23.8)		
4–6 times/week	11 (2.7)	14 (1.5)		
1–3 times/week	78 (19.1)	157 (16.6)		
< 1 time/week	224 (54.8)	548 (58.1)		
**Eggs (%)**			0.04	0.472
≥ 1 time/day	231 (56.5)	512 (54.2)		
4-6times/week	51 (12.5)	101 (10.7)		
1-3times/week	87 (21.3)	232 (24.6)		
< 1time/week	40 (9.8)	99 (10.5)		
**Legumes (%)**			0.06	0.195
≥ 1 time/day	9 (2.2)	24 (2.5)		
4–6 times/week	7 (1.7)	37 (3.9)		
1–3 times/week	198 (48.4)	453 (48.0)		
< 1 time/week	195 (47.7)	430 (45.6)		
**Nuts (%)**			0.04	0.490
≥ 1 time/day	41 (10.0)	76 (8.1)		
4–6 times/week	31 (7.6)	69 (7.3)		
1–3 times/week	72 (17.6)	192 (20.3)		
< 1time/week	265 (64.8)	607 (64.3)		
**Fish (%)**			0.07	0.074
≥ 1 time/day	17 (4.2)	42 (4.4)		
4–6 times/week	60 (14.7)	157 (16.6)		
1–3 times/week	129 (31.5)	349 (37.0)		
< 1 time/week	203 (49.6)	396 (41.9)		
**Poultry (%)**			0.03	0.765
≥ 1 time/day	3 (0.7)	7 (0.7)		
4–6 times/week	3 (0.7)	4 (0.4)		
1–3 times/week	89 (21.8)	225 (23.8)		
< 1 time/week	314 (76.8)	708 (75.0)		
**Red meat (%)**			0.03	0.721
≥ 1 time/day	27(6.6)	74(7.8)		
4–6 times/week	17(4.2)	32(3.4)		
1–3 times/week	163(39.9)	388(41.1)		
< 1 time/week	202(49.4)	450(47.7)		
**Processed meat (%)**			0.05	0.234
≥ 1 time/day	0 (0.0)	0 (0.0)		
4–6 times/week	1 (0.2)	0 (0.0)		
1–3 times/week	19 (4.6)	36 (3.8)		
< 1 time/week	389 (95.1)	908 (96.2)		
**Dairy products (%)**			0.04	0.513
≥ 1 time/day	90 (22.0)	176 (18.6)		
4–6 times/week	13 (3.2)	27 (2.9)		
1–3 times/week	31 (7.6)	79 (8.4)		
< 1 time/week	275 (67.2)	662 (70.1)		
**Alcohol (%)**			0.04	0.184
≥ 1 cup/week	77 (18.8)	208 (22.0)		
< 1 cup/week	332 (81.2)	736 (78.0)		
**Tea (%)**			0.01	0.910
≥ 1cup/week	67 (16.4)	157 (16.6)		
< 1cup/week	342 (83.6)	787 (83.4)		

*TRF* Time restricted feeding.

*TRF is the control group. ^△^*φ* indicates effects sizes in Chi-squared test.

### Demographic, psychosocial and behavioral factors

Table 3 shows the PRs for participation in TRF group according to demographic, psychosocial and behavioral factors. In the adjusted Model 1, older age, hypertension and stroke were positively associated with participation in TRF group, while farming and night-time feeding were negatively correlated with participation in TRF group (all *P* < 0.05).

**Table 3:** Crude and adjusted analyses of the association between TRF (time feeding restricted to 10 h vs. no restriction) and demographic, psychosocial and behavioral factors.

	n (%)	Crude analysis PR (95% CI)	Model 1^a^ PR (95% CI)
**Age group (years)**			
60–64	47 (3.5)	Reference	Reference
65–74	823 (60.8)	1.09 (0.61–1.94)	1.11 (0.62–2.00)
≥ 75	483 (35.7)	1.37 (0.76–2.46)*	1.30 (0.71–2.39)*
**Sex**			
Male	563 (41.6)	Reference	Reference
Female	790 (58.4)	1.10 (0.90–1.34)	1.12 (0.88–1.43)
**Illiterate**			
No	1182 (87.4)	Reference	Reference
Yes	171 (12.6)	1.24 (0.94–1.62)	1.17 (0.87–1.58)
**Widowed**			
No	1076 (79.5)	Reference	Reference
Yes	277 (20.5)	1.14 (0.91–1.44)	1.03 (0.70–1.50)
**Living alone**			
No	1115 (82.4)	Reference	Reference
Yes	238 (17.6)	1.09 (0.85–1.39)	1.06 (0.73–1.55)
**Farming**			
No	585 (43.2)	Reference	Reference
Yes	768 (56.8)	0.84 (0.69–1.02)*	0.76 (0.62–0.93)*
**Smoking**			
Non-smoker	945 (69.8)	Reference	Reference
Former smoker	219 (16.2)	1.16 (0.86–1.55)	1.13 (0.78–1.65)
Current smoker	189 (14.0)	0.93 (0.64–1.36)	0.89 (0.61–1.31)
**Drinking**			
Never drinker	850 (62.8)	Reference	Reference
Former drinker	143 (10.6)	0.91 (0.60–1.37)	0.88 (0.58–1.33)
Occasional drinker	151 (11.2)	1.29 (0.88–1.87)	1.29 (0.87–1.90)
Current drinker	209 (15.4)	1.11 (0.84–1.48)	1.01 (0.73–1.39)
**The time of food intake**			
Daytime feeding	1196 (88.4)	Reference	Reference
Night-time feeding	157 (11.6)	0.43 (0.31–0.96)*	0.41 (0.26–0.64)*
**Type-2 diabetes**			
No	1119 (82.7)	Reference	Reference
Yes	234 (17.3)	0.94 (0.72–1.22)	0.84 (0.64–1.11)
**Hypertension**			
No	498 (36.8)	Reference	Reference
Yes	855 (63.2)	1.25 (1.02–1.54)*	1.19 (0.95–1.48)*
**Dyslipidemia**			
No	1175 (86.8)	Reference	Reference
Yes	178 (13.2)	1.10 (0.83–1.44)	1.06 (0.80–1.42)
**Stroke**			
No	951 (70.3)	Reference	Reference
Yes	402 (29.7)	1.21 (0.98–1.48)*	1.09 (0.87–1.36)*
**Heart disease**			
No	879 (65.0)	Reference	Reference
Yes	474 (35.0)	1.02 (0.83–1.25)	1.04 (0.84–1.29)
**Cancer**			
No	1307 (96.6)	Reference	Reference
Yes	46 (3.4)	0.86 (0.48–1.53)	1.01 (0.57–1.82)

Poisson regression, TRF is the dependent variable.

*TRF* Time restricted feeding, *PR* prevalence ratio, *CI* confidence interval.

^a^Model 1 is adjusted for age, sex, BMI, IADL, illiterate, widowed, living alone, farming, smoking, drinking, the time of food intake, IPAQ, type-2 diabetes, hypertension, dyslipidemia, stroke, heart disease, and cancer.

Likelihood ratio test, all *P* < 0.001; *P < 0.05.

### The relationship between TRF and cognitive status

**Figure 1:**
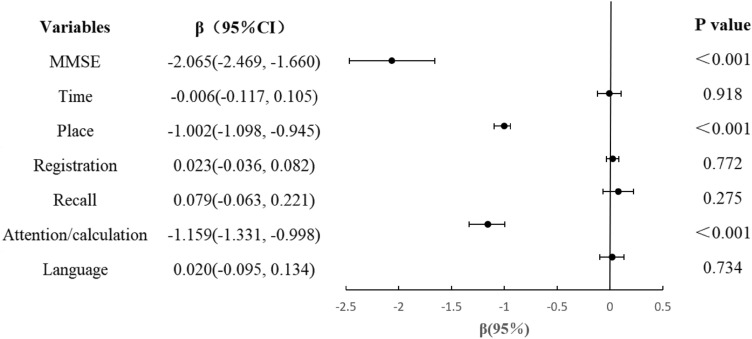
Linear regression between time restricted feeding and cognitive status in the study sample (time feeding restricted to 10 h vs. no restriction). *Multiple linear regression model, with cognitive status as the dependent variable. *CI* confidence interval. The results are adjusted for age, sex, BMI, IADL, illiterate, widowed, living alone, farming, smoking, drinking, night-time feeding, IPAQ, type-2 diabetes, hypertension, dyslipidemia, stroke, heart disease, and cancer. *MMSE Mini-mental State Examination.

Figure 1 shows a linear regression between TRF and MMSE domains of CI. We used "Orientation to time", "Orientation to place", "Registration", "Attention/calculation", "Recall" and "Language" as the dependent variables in the regression model to further explore the specific impact of TRF on cognitive function. After adjusting for covariates (age, male, BMI, IADL, illiterate, widowed, living alone, farming, smoker, drinker, night-time feeding, IPAQ, type-2 diabetes, hypertension, dyslipidemia, stroke, heart disease, and cancer), We found that TRF affected negatively the "Orientation to place" (*P* < 0.001) and "attention/calculation" (*P* < 0.001) functions of six cognitive domains.

## Discussion

The purpose of our study is to investigate the relationship between TRF and CI, and the impact of TRF on different cognitive domains of the elderly in Chinese community. Our cross-sectional study results suggested that TRF may be associated with a higher risk of CI, and affect the performance of elderly people in orientation to place and attention and calculation. We explored which cognitive domains on the MMSE are more strongly associated with TRF, in which TRF was negatively related to the "Orientation to place" and "attention/calculation" functions. As far as we know, this is the first study to explore the different relationship in TRF and specific cognitive domains in older Chinese community-dwelling adults. In addition, we also analyzed the associations between potential correlates and the participation in TRF groups. The results showed that older age, hypertension and stroke were positively associated with participation in TRF group, while farming and night-time feeding were negatively correlated with participation in TRF group.

Our study shows a significant correlation between TRF and MMSE (the evaluation indicators of cognitive function in this study), which still exists after adjusting for confounding factors. Some studies have found that TRF, which may be associated with increased synaptic plasticity and increased production of new neurons from neural stem cells, inducing several molecular and cellular adaptations in neurons^21^. Overall, this enhances cellular stress resistance, synaptic plasticity, and neurogenesis, so as to influence neurogenesis and cognitive function^22^. These mechanisms have proved the positive effect of TRF on cognitive status. However, in the results of this study, individuals having TRF were more likely to have CI and TRF may be negatively related to attention/calculation function (All *P* < 0.001). At present, few studies have explored the impact of TRF on specific cognitive domains. The ability to maintain attention and spatial orientation (SO) is essential. Attention is closely associated with impairment of other cognitive functions, and the deterioration of SO in MCI is related to a higher risk of progression to AD^1^. In the process of healthy aging, people are less able to maintain their attention with the increase of age, and there are more and more compensatory interactions between attention network and the emergence of clinical symptoms of MCI, which may no longer be effective^1^. Due to the complex mechanism, the specific effect of fasting time on the nervous system has not been determined. Although our study design does not discuss the causal relationship between TRF and CI, based on the existing research results, we speculate that TRF and CI are mutually causal. In the mouse model experiment, Andika and colleagues found that IF can alleviate hippocampal neuronal loss and restore the cognitive function, but it will enhance astrocytosis^23^. Astrocytosis is characterized by the increase of GFAP homologous cells in the brain. After the IF intervention, a further hippocampal GFAP expression is elevated, which induces a sign of worsening neuropathology^23^. This may explain the results of this study in some ways. However, so far, the specific role that astrocytes may play has not been explored. In addition, long-term time restrictions on eating may potentially expose people to hunger, irritability, and reduced ability to concentrate^21^. Therefore, TRF may be one of the reasons for the change of cognitive outcomes. It is necessary to explore the impact of TRF on various areas of cognitive function. In addition, CI may affect patients' eating habits and change their daily meal time. Some studies have found that the decline of cognitive ability, memory, learning disabilities, attention deficits can all lead to changes in patients' regular eating behavior, reduced necessary nutrition intake, and patients may refuse to accept nutritional interventions^24–26^. At the same time, the change of eating behavior will affect the disease status of the elderly by affecting the intake of nutrients, increase the risk of disease in the elderly, and cause further harm to the cognitive status of the elderly^25^. In future studies, intervention study and randomized controlled trials in subjects at an initial stage of MCI should also be carried out, in order to understand the long-term impact of TRF and its causal relationship with CI.

In this study, about 30.2% of participants showed TRF. We speculate that this may be related to the older average age of the participants in this study. According to the sleep habits of the elderly in China, older people tend to fall asleep earlier. In our research results, the average sleep duration of the elderly is 8.42 ± 1.34 h, and 46.5% of the elderly are used to falling asleep before 8 p.m. The shorter daytime activity time may indirectly lead to the shorter eating time window. Moreover, we also speculate that the age of participants may be one of the factors affecting the cognitive results of TRF. Because age is the main unalterable risk factor for cognitive impairment^2^. The older the age, the greater the risk of cognitive impairment. In our study, the age of participants in the TRF group were older (*P* = 0.017), with an average age of 73.86 ± 6.35. In our regression model, older age was positively associated with participation in TRF group (*P* < 0.05). Several studies have shown that the increase of age may lead to an increase in health risks such as weakness and malnutrition, strict dietary restriction may not be suitable for frail older adults at risk of malnutrition, hypothermia, and bacterial/viral infection^11,12^. Brandhorst et al.^15^ pointed out that the 65–70 age range represents a key transition point, and dietary interventions in rodents and humans must be modified to optimize efficacy in at least two adult age ranges. Some studies tried to provide an explanation for the uneven tendency of age-related brain changes between older adults through cognitive reserve (CR)^27^. They believed that individuals with higher reserve levels were more successful in coping with brain injury than those with lower reserve levels, so the increase of cognitive reserve level might lead to the decline of deterioration process^27^. In addition, it is particularly important to understand the age-specific effects of these interventions, considering the major changes in weight, growth hormones, and steroid hormones that occur at different ages, and the studies indicating that certain restriction can be beneficial or have no negative effects in young, but may have effects on old organisms^16^.

In our study population, the cross-sectional data showed that the average IADL score of TRF group was 6.57 ± 1.14, which was lower than that of non-time limited group (*P* = 0.021). At present, there are few studies directly involving the relationship between TRF and IADL, which is closely related to cognitive status. Some studies have pointed out that there was no significant changes in activity at the end of TRF compared to baseline, but there was a trend toward a reduction in physical functional activities^28^. While the change of IADL is most significant in populations with dementia, studies also showed that variation in IADL was also observed in the MCI population^29^. In other words, even if someone maintains relatively complete functional independence, subtle changes in functional proficiency can be reflected in IADL assessment. IADL may precede the development of cognitive impairment, so it can be used as an important risk marker for clinical diagnosis of dementia. A longitudinal survey of people aged 65 and older without dementia found that a point of reduced physical function was associated with an increased risk of dementia, Alzheimer's disease and an increased rate of cognitive decline during 6-year follow-up^30^. In addition, Kwak and his colleagues found that more than half of the effects of brain structure on IADL were mediated by the combined score of cognitive and behavioral scores^31^. The assessment items of IADL may be altered by the variety of daily activities and the difference across the lifestyle context of individuals^29^. The old adults with good lifestyle and larger protective factors would delay or buffer neuropathy, resulting in the predictive gap and deviation of IADL. Therefore, future studies need to evaluate the accuracy of prediction models of IADL in different clinical populations with unified predictors in order to better screen the risk of cognitive impairment^28^. At the same time, it is also necessary to explore the relationship between daily functional activities and dietary behavior, including dietary window and food group, in order to explore the relevant mechanism.

Furthermore, our study also found that the elderly in the TRF group have a higher proportion of hypertension and stroke (*P* = 0.012; *P* = 0.033, respectively), which may also be one of the potential factors leading to a higher risk of cognitive impairment in the TRF group. The results of Poisson regression also showed that hypertension and stroke were positively associated with participation in TRF group (*P* < 0.05, respectively). In previous studies, some scholars pointed out that due to the lack of human research related to TRF, the evidence supporting fasting as a treatment in human neurological disorders, including neurodegeneration, stroke, epilepsy, and multiple sclerosis, might be indirect or non-existent^32^. Therefore, the high proportion of stroke history in TRF group in this study is not unreasonable, and fasting is not necessarily beneficial to stroke. Furthermore, circadian rhythm is also one of the key factors affecting the results of TRF. The results of TRF in human seem to depend on the time of the eating window of the day, not just on the fasting time itself^33^. Our research also confirm this point. In our research results, we found that the number of agricultural workers in the in the No restriction group was higher, and farming was negatively correlated with participation in TRF group. It is speculated that due to occupation, agricultural workers often need to get up earlier than others. In our research results, 87.9% of agricultural workers get up at about 5 a.m. or earlier, but their sleep time is the same as that of other occupations. Therefore, this part of the population takes their first meal earlier, but their dinner time is not affected, resulting in their eating time window exceeding 10 h. Although different occupations cause changes in eating time, the results of the FFQ showed that there was no significant difference in dietary intake frequency between the TRF group and no restriction group. TRF only limited the time window of eating without changing the frequency of participants' specific dietary intake and eating habits. The time of food intake may change the metabolic response to TRF intervention, and metabolic syndrome is a major risk factor to change cognitive outcomes^34,35^. In this study, most participants used to daytime feeding, while in the TRF group, the proportion was much higher (*P* < 0.001). For the fasting time of TRF, although it usually does not lead to ketosis, it is sufficient to stimulate many of the pathway related to long-term fasting approach, such as autophagy^36^. In the heart and blood vessels, autophagy plays a fundamental role not only in the process of cardiac embryonic development, but also in normal cardiovascular function. It is suggested that many peptides and hormones involved in cardiovascular physiology are also regulated by autophagy^37^. Therefore, it is speculated that dysregulation of autophagy may be related to hypertension, obesity, diabetes and terminal organ damage^38^. This injury may further lead to the damage of TRF to cognitive function. Moreover, a potential confusion in current TRF studies is the lack of an objective measure to assess eating time. Although FFQ can effectively evaluate the recent dietary frequency and food ratio of subjects, but self-reporting of eating duration may not be optimal^39^. Since the concept of limiting eating time has only recently emerged, the method of objectively recording eating time has not been well optimized^40^. These problems also need to be further explored and standardized in future research.

### Strengths and limitations

This study has a major strength to be the first reporting an association between TRF and specific cognitive domains of elderly people over 60 years old in suburban areas of China, which was not included in previous studies, revealing the possible adverse effects of TRF on cognitive function, which indicated that this hypothesis to be further tested in future studies. In addition, we also discussed the factors that may affect the results of TRF, such as the age of participants, cardiac metabolic diseases, circadian rhythm of eating, etc. However, in light of some limitations, the results of this study should be further considered. Firstly, since this study involves Chinese elderly over 60 for the first time, considering the possible negative impact of TRF on the health of the elderly, more baseline data are still needed before the intervention experiment, so we only evaluated the baseline data and did not conduct the clinical intervention experiment. In fact, it is crucial to master the preliminary data before establishing the intervention test, which will strongly affect the eating habits of older adults at risk of cognitive impairment and altered cognitive function (lack of compliance). Another limitation is that because we recorded dietary intake frequency in the past month only from the self-report of participants, there is a certain degree of subjective bias, which would cause some confounding effects on the results. Finally, although we found the association between TRF and some specific cognitive domains which was statistically significant, the intensity of these findings should be confirmed in future studies, and there should be larger samples, more cases and more individuals exposed to the variables of interest.

## Conclusions

In conclusion, among Chinese elderly over 60 years old, restricting the daily time feeding window may have a negative effect on cognitive status. In addition, TRF is negatively related to "Orientation to place" and "Attention/calculation" function. In the future, it is necessary to conduct larger-scale and longer-term intervention trials to investigate the long-term impact of TRF on CI and different cognitive domains, so as to formulate targeted nutrition intervention policies and promote the healthy aging of the elderly.

## Data Availability

All data generated or analysed during this study are included in this published article.
